# Description of the methodology used in an ongoing pediatric care interventional study of children born with cleft lip and palate in South America [NCT00097149]

**DOI:** 10.1186/1471-2431-6-9

**Published:** 2006-03-24

**Authors:** George L Wehby, Eduardo E Castilla, Norman Goco, Monica Rittler, Viviana Cosentino, Lorette Javois, Ann Marie McCarthy, Georgiy Bobashev, Stephen Litavecz, Alejandra Mariona, Graca Dutra, Jorge S López-Camelo, Iêda M Orioli, Jeffrey C Murray

**Affiliations:** 1 Department of Health Management and Policy, University of Iowa, Iowa City, USA; 2 Latin American Collaborative Study of Congenital Malformations (ECLAMC), Centro de Educación Médica e Investigaciones Clínicas (CEMIC), Buenos Aires, Argentina; 3 ECLAMC, Instituto Oswaldo Cruz, Rio de Janeiro, Brazil; 4 RTI International, Research Triangle Park, USA; 5 ECLAMC, Maternidad Ramón Sardá, Buenos Aires, Argentina; 6 Center for Developmental Biology and Perinatal Medicine, National Institute of Child Health and Human Development, Bethesda, USA; 7 College of Nursing, University of Iowa, Iowa City, USA; 8 ECLAMC, Instituto Multidisciplinario de Biologia Celular (IMBICE), La Plata, Argentina; 9 ECLAMC, Departamento de Genética, Universidade Federal do Rio de Janeiro, Rio de Janeiro, Brazil; 10 Department of Pediatrics, University of Iowa, Iowa City, USA

## Abstract

**Background:**

The contribution of birth defects, including cleft lip and palate, to neonatal and infant mortality and morbidity is substantial. As other mortality and morbidity causes including infections, hygiene, prematurity, and nutrition are eradicated in less developed countries, the burden of birth defects will increase proportionally.

**Methods/Design:**

We are using cleft lip and palate as a sentinel birth defect to evaluate its burden on neonatal and infant health and to assess the effectiveness of systematic pediatric care during the first month and first two years of life in decreasing this burden. The neonatal intervention, consisting of weekly pediatric evaluation and referral to appropriate care, is delivered to about 696 infants born with cleft lip and/or palate in 47 hospitals in South America. Neonatal mortality in this group will be compared to that in a retrospective control group of about 464 infants born with cleft lip and/or palate in the same hospitals. The subgroup of infants with isolated clefts of both the lip and palate (about 264) is also randomized into two groups, intervened and non-intervened, and further followed up over 2 years. Intervened cases are evaluated by pediatricians every three months and referred for appropriate care. The intervened and non-intervened cases will be compared over study outcomes to evaluate the intervention effectiveness. Non-intervened cases are matched and compared to healthy controls to assess the burden of cleft lip and palate. Outcomes include child's neurological and physical development and family social and economic conditions.

**Discussion:**

Large-scale clinical trials to improve infant health in developing countries are commonly suggested, making it important to share the methods used in ongoing studies with other investigators implementing similar research. We describe here the content of our ongoing pediatric care study in South America. We hope that this may help researchers targeting this area to plan their studies more effectively and encourage the development of similar research efforts to target other birth defects or infant outcomes such as prematurity and low birth weight.

## Background

Neonatal and infant mortality and morbidity are high in less developed countries. Each year about 10 million children die worldwide, with extensive between and within country variation [1,2]. Almost half of these deaths (about 4 million) occur among newborns before one month of age [2-4]. The majority (about 95%) of all neonatal deaths occur in less developed countries, where 34 neonates die among every 1000 live births, compared to 5 neonates in developed countries [3,4]. In the past two decades, greater reductions have been observed in child mortality than in neonatal mortality with the lowest reductions observed in the early neonatal phase [4], leading to a greater number of deaths within the first month of life.

Among the main causes of neonatal mortality in developing countries (infections, prematurity complications, and birth asphyxia), congenital anomalies constitute the fourth leading cause, and are responsible for about 7–10% and 3.8% of neonatal and under – 5 mortality, respectively [1,3,4]. Congenital malformations are increasingly contributing to overall infant mortality in developed countries such as the United States [5], where 20% of infant deaths in 2001 were related to congenital malformations [6]. The burden of birth defects will also be expected to increase in developing countries as public health, nutrition, and other primary health interventions succeed in reducing main mortality and morbidity causes such as infections and low birth weight. Besides mortality, birth defects also increase the risk for disability. In 2002, more than 27 million lost Disability Adjusted Life Years (DALYs) forming about 1.8% of the overall burden of disease were related to congenital abnormalities [1].

Oral clefts are common birth defects that affect about 1 in every 700 births with varying prevalence by population origin and socioeconomic status [7,8]. Cleft lip and palate occurs as both isolated and syndromic forms [9]. Isolated forms are unassociated with any other structural or cognitive birth defects. Syndromic forms of clefts have a wide range of etiologies with more than 400 reported in Online Mendelian Inherited Diseases in Man [10] from single gene causes and with other clefts occurring secondary to chromosomal anomalies, teratogenic exposures, and as sporadic disorders without recognized etiologies.

Isolated forms are readily targeted by care provided by multi-specialty teams yet may still have substantial infant morbidity and mortality specifically in cases with limited access to care that might include for example, education for appropriate feeding. Malnourishment of infants born with clefts because of feeding problems may enhance the impact of other mortality risk factors such as infections. A few studies have reported several-fold increased neonatal and infant mortality risks among infants born with clefts, specifically among syndromic forms [11-13]. Further, there is evidence of a long-term burden even of isolated forms, mainly in challenges for psychological and social adjustment [14-16] as well as reduced life expectancy [17].

Since clefts are readily apparent at birth, they can serve as sentinels for other birth defects and their impact. We are studying cleft lip and palate as a model birth defect to determine whether an increased pediatric care model can decrease mortality and morbidity in an infant population born in-hospital mainly to indigent populations in South America. The primary research aims include evaluating if neonatal mortality among children born with cleft lip and/or cleft palate can be decreased by a systematic pediatric intervention over the first month of life, and if systematic pediatric care over the first two years of life can improve the neurodevelopment and growth of children born with isolated forms of cleft lip with cleft palate compared to usual pediatric care. The neurodevelopment and growth of this latter group will also be compared to those of healthy control babies without birth defects receiving usual pediatric care. Secondary research aims include evaluating the effectiveness of the pediatric intervention and follow-up program on other neonatal and two-year of life health outcomes, which are described below in further detail.

The study benefits from a well-established consortium of pediatricians working under the auspices of the Estudio Colaborativo Latino Americano de Malformaciones Congenitas (ECLAMC), a birth defects surveillance program that has been active in South America since 1967 [18]. Currently, about 80 hospitals are enrolled in ECLAMC and carry out surveillance of birth defects on approximately 200,000 births per year. We used this infrastructure to identify pediatricians who could provide direct care to infants born with cleft lip and palate and follow them closely for the first month to ensure that appropriate medical and surgical interventions were undertaken. The impact of systematic pediatric care throughout the first two years of life on health is also being studied in the subgroup with isolated cleft lip and cleft palate using the same infrastructure.

The objectives of this paper include reporting how the project was established and describing study design and interventions. There has been increased emphasis recently for establishing large-scale clinical trials to study perinatal and neonatal health interventions in less developed countries. Recent study reviews have identified the need for more clinical trials to address several research gaps in the provision of neonatal care in these settings [19,20]. Further evaluation of the effectiveness of alternative models for neonatal care content and delivery is one emphasized theme. Yet little empirical guidance on how to optimally design and implement these trials in less developed countries is available. As more resources are being allocated into this field, it is important to share methods of ongoing large-scale interventional trials. This may help researchers plan their studies to appropriately adjust interventions, improve procedures, and estimate adequate budgets. We hope that sharing our study methods would be of direct relevance to researchers and funding agencies targeting this area.

## Methods/Design

### Setting and participants

Study participants are being recruited in 47 ECLAMC hospitals from 35 cities in Argentina, Brazil, Bolivia, Chile, Colombia, Ecuador and Venezuela (see list in acknowledgements section). Study participants include live born infants with cleft lip and/or palate and healthy infants and their parents. Infants with a typical oral cleft as the only detected congenital anomaly are considered isolated cases. Infants with atypical oral clefts (oblique facial clefts, congenital healed clefts, midline cleft lip, submucous cleft palate, bifid uvula) are excluded. In this study, syndromic forms of cleft lip and palate include cases with recognized syndromes, cases with chromosome abnormalities, cases with one or more major structural anomalies other than cleft lip and palate, cases with cognitive delay (IQ or equivalent less than 80), or cases exposed to recognized teratogens in utero (phenytoin or valproic acid). Cases born to mothers who smoked or used alcohol during pregnancy who otherwise do not meet the syndromic form criteria listed above are considered isolated cases. Syndromes are classified using chromosomal analysis and/or by physical findings, and all cases are reviewed by at least two experienced dysmorphologists [Eduardo Castilla, Monica Rittler, Viviana Cosentino, Iêda Orioli, Jeff Murray].

#### Inclusion/exclusion criteria

**Figure 1:**
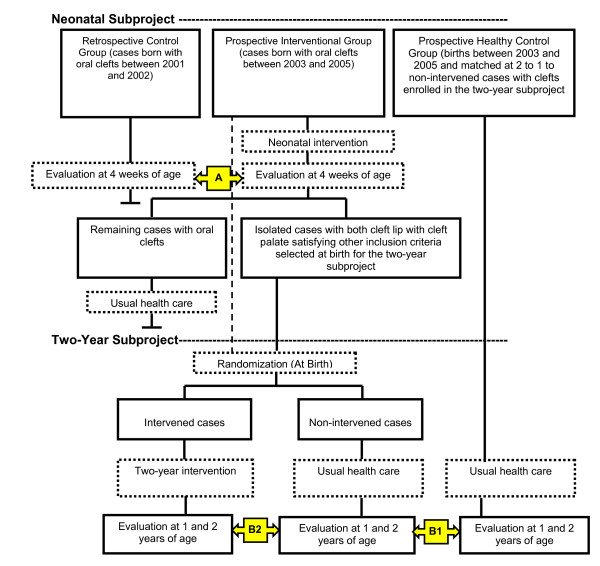
**Study Design**. Figure 1 presents a flowchart of the study design of the neonatal and the two-year subprojects. All infants born with a typical form of cleft lip and palate in the study hospitals are enrolled in the neonatal subproject. Only isolated cases with both cleft of lip and cleft palate who are singleton births, normal birth weight, and without complications other than cleft lip and palate that require systematic care are enrolled at birth in the two-year subproject and randomized into intervened and non-intervened groups. A group of healthy controls is matched to the non-intervened group by gender, date and hospital of birth at a ratio of 2 to 1. A = Evaluating the impact of the intervention on neonatal mortality. B1 = Evaluating the impacts of cleft lip and palate on child's development and family socioeconomic outcomes (Two-year subproject). B2 = Evaluating the impact of the two-year intervention on child development and family socioeconomic outcomes.

The neonatal prospective group that receives the pediatric intervention includes infants born with typical clefts of any etiology or affected segment (lip, gum, hard palate, soft palate) in participating hospitals between January, 2003 and December, 2005, and diagnosed as eligible within the first 48 hours after birth, with an initially projected total of about 696 cases to be available for recruitment. No other inclusion or exclusion criteria are imposed on this group. Since it was considered unethical to randomize neonatal subjects into treatment and no treatment control groups, as there was a strong sentiment that the intervention forms the current standard of care in the United States, a retrospective control group born in 2001 and 2002 with clefts in the same hospitals was chosen. This control group is projected to include about 464 cases, yet data are still being processed. This group of infants had previously received routine pediatric and medical care offered by their communities during the neonatal period. A schema of the study design is shown in Figure 1.

The two-year study group includes children from the neonatal prospective subgroup who are born with isolated forms of both cleft lip and cleft palate. Children with syndromic forms are excluded, as are twins and children with birth weight less than 2500 grams or who have complications other than cleft lip and palate that require systematic care. A group of healthy controls born without birth defects and subject to the same exclusion criteria and whose gestational age is between 37 and 42 weeks are also included in the two-year study.

### Neonatal intervention

The neonatal intervention in the prospective group involves weekly monitoring and evaluation of infant health through follow up visits to participating ECLAMC pediatricians. The visits usually occur at the hospital of birth or private offices of participating pediatricians; visits to subjects' homes are encouraged when the weekly visits are missed. During each of the four neonatal visits extending to one month of age, the pediatrician conducts a clinical assessment of infant health and growth. Special emphasis is placed on weight gain, feeding problems, and medical complications such as hyperbilirubinemia or infection. The pediatrician also interviews parents for family and household factors that may affect the infant health. Based on assessment results, the pediatrician refers the infant to appropriate health professionals for specialized care. Recommendations and instructions to parents regarding infant needs and optimal care are also provided. If the child demonstrates a failure to gain weight, physicians will, using standard medical judgment, determine whether hospital admission is appropriate or whether the child can be sent home. If sent home, the infant may be followed at more frequent intervals depending on the severity of the weight loss or other medical complications. Any decrease in weight by greater than 10 percent will require hospital admission and weight decreases between 0 and 10 percent will have suitability for admission determined by the referring physician.

The impact of the pediatric intervention on neonatal mortality will be assessed by comparing mortality rates between the retrospective and prospective study groups. Birth time periods for the two groups are close and consecutive, limiting time changes in conditions such as quality of health care that may affect mortality and confound the evaluation of the intervention's impact.

ECLAMC started in 2001 a program titled "ORIENT" to provide guidance prior to hospital discharge to parents of infants born with birth defects about appropriate care and available treatments for the infant [18]. The retrospective group has also received this standard of care available at ECLAMC hospitals in addition to the standard of care available in the community.

### Two-year intervention

A second intervention is being carried out on a subset of children with isolated cleft lip with cleft palate, with an initially projected total of about 264 cases to be available for this subproject. Screening for eligibility and enrollment into the two-year subproject occurs at birth. Eligible children with clefts are randomized at birth into intervened or non-intervened groups. The pediatricians conduct a thorough clinical assessment of the development of intervened children every three months up to two years of age, including evaluation of physical growth (height, weight, and head circumference), neurodevelopment, using the Bayley Infant Neurodevelopmental Screener (BINS), and language, using the Receptive-Expressive Emergent Language Test (REEL). The BINS is used to monitor the course of an infant's development and identifies infants with developmental delays or neurological impairments who are in need of further diagnostic evaluation [21,22]. The REEL screens for delays in emergent speech and language development in medically and environmentally at-risk children [23,24]. The pediatricians also perform a dental assessment for the child and record erupted teeth and any abnormalities, check the nutritional status including feeding content, process, and problems, and inquire about all child morbidities and treatments since the last evaluation and overall changes in health and socioeconomics of family members (e.g. parental illnesses, changes in employment and marital status of the parents, and changes in schooling and health status of siblings). The pediatricians refer the intervened children to specialized health facilities and professionals based on their clinical assessments and the practice standards available for dealing with clefts and emphasize to parents the importance of compliance with these referrals. The pediatricians also provide counseling to parents in order to maintain a healthy and loving familial environment that provides continuous care to the child.

The healthy control children enrolled in the two-year study as a normal control group are matched to the non-intervened cleft group two to one at birth for sex, hospital, and week of birth. The non-intervened control group of children with clefts and the healthy control group receive the standard of care available in their respective community according to parental wishes. Both control groups are assessed at one and two-year follow-ups to measure study outcomes. The normal control group will be compared to the non-intervened cases with clefts to assess the impact of clefting on child health and family socioeconomic outcomes. The intervened and non-intervened cases with clefts will be compared to evaluate the effectiveness of the intervention in lowering the burden of clefting on the child and the family (see Figure 1).

#### Randomization procedures in the two-year study

The randomization of cases in the two-year study into intervened and non-intervened groups is based on a randomization sequence generated by the Data Center at the Research Triangle Institute (RTI) International, and is stratified by participating hospitals. The Data Center provides each pediatrician a set of sealed envelopes that contain a sheet indicating treatment assignment. The pediatrician administers the randomization procedure by assigning the next available envelope as each case is enrolled into the study. Study personnel and subjects are not blinded to the randomization assignments, but safeguards are in place to ensure that assignments are not known a priori. The pediatrician reports within 24 hours of each randomization to the Data Center the subject ID, the tracking number of the randomization envelope used, and the group assignment for verification, and any inconsistencies are readily corrected. The randomization sequence lists are kept in secure locations at the Data Center and ECLAMC headquarters.

### Study outcomes

The neonatal outcomes include overall mortality at 28 days of life (primary outcome), mortality in subgroups of isolated and non-isolated cleft groups, hospitalization days, and refinement of syndromic classification in the retrospective group. The impact of the neonatal intervention on infant mortality will also be evaluated and phone interviews are conducted with parents of prospective and retrospective groups when needed to inquire about child's survival at one year of age. The primary outcomes of the two-year study include overall neurodevelopment (measured by BINS) and weight changes. Secondary outcomes include performance on four ability areas including neurological functions/intactness, receptive functions, expressive functions, and cognitive processes (measured by the BINS), speech (measured by REEL), height changes, hearing, timing of cleft surgery, mortality, refinement in syndromic status classification, and emotional, social, and economic performance of the family.

### Power analysis

The power analyses prepared prior to study initiation are reported in Tables 1 and 2 for the neonatal and the two-year subprojects respectively. The power estimation for the primary hypothesis of reduction in neonatal mortality in the prospective group compared to the retrospective control group assumes a simple random sample scheme and a probability of Type I error of 0.05. Power is estimated for alternative neonatal mortality rates in both the prospective and retrospective control groups due to the lack of robust estimates at the time of study initiation of baseline mortality rates in the retrospective group and of potential achievable reductions in neonatal mortality due to the intervention. Mortality rates of 20, 25, and 30% are assumed for the retrospective group. The neonatal mortality rate in the prospective group is also assumed to not exceed 25% and to be not higher than that of the retrospective group (one-sided test). The power calculation for the neonatal subproject is based on a sample size of 464 cases in the retrospective group and 696 cases in the prospective group. This analysis shows that acceptable power (>0.7) is available for reductions of about 30% or more in assumed baseline neonatal mortality rates (See Table 1).

**Table 1:** Power to Detect Differences in Neonatal Mortality Proportions

*Neonatal Mortality in Retrospective Group*	*Neonatal Mortality in Prospective Group*	*Power*
0.20	0.12	0.97
	0.15	0.68
	0.17	0.34
0.25	0.15	0.99
	0.17	0.94
	0.20	0.61
	0.22	0.30
0.30	0.15	>0.99
	0.17	>0.99
	0.2	>0.99
	0.22	0.98

**Table 2:** Power to Detect Differences in Means of Continuous Outcomes in the Two-Year Study

Sample Size per Group	Standardized Effect Size			
	0.15	0.25	0.4	0.5
60	0.30	0.60	0.92	0.98
90	0.40	0.76	0.98	>0.99
120	0.49	0.85	>0.99	>0.99
150	0.55	0.91	>0.99	>0.99
160	0.59	0.93	>0.99	>0.99
170	0.61	0.94	>0.99	>0.99

The power analysis to detect differences in means of the continuous outcomes of the two-year study (e.g. BINS and REEL scores or weight gain) between the study groups is also based on alternative effect sizes due to limited information available a priori on these parameters and on alternative sample sizes. In this analysis, power estimation assumes a paired sample where one subject is selected at random and another is a matched control (correlation of 0.5 between the subjects in a matched pair). Power is evaluated for standardized effect sizes (i.e. mean difference divided by standard deviation) of 0.15, 0.25, 0.4, and 0.5, using a Type I error probability of 0.05 and a one sided test. The analysis shows that acceptable power (>0.7) is available to detect a standardized effect size of 0.25 or higher with a sample size of 90 cases per study group. As an example, a 0.25 standardized effect size for the receptive language quotient of the REEL measure is equivalent to a 4-point difference from a mean score of 108 with a standard deviation of 16, which are reference estimates for samples of normal babies under 36 months of age.

### Statistical analysis

The effects of the pediatric interventions on the studied outcomes will be analyzed using simple statistical tests for comparisons between intervened and control groups as well as multivariate regression techniques that account for potential confounders between the evaluated groups such as cleft type, baseline health characteristics, family socioeconomic status, and for sample clustering across hospitals and countries of birth. Logistic regression and Cox-proportional hazard models will be used to evaluate differences in overall neonatal mortality between the prospective and retrospective groups as well as in subgroups of isolated and non-isolated clefts. Similarly, weight changes and other developmental (e.g. BINS, REEL, height, etc.) and socioeconomic outcomes (e.g. maternal employment) will also be compared between the intervened and non-intervened cleft groups on one side and between the non-intervened cleft group and the healthy control group on another side using regression analyses. Correlation among subjects recruited within the same hospital (within hospital clustering) and within-subject correlation in analyses involving multiple observations per subject will be accounted for by using hierarchical/mixed models or Generalized Estimating Equations (GEE) methods or by applying robust estimators for the variance-covariance of regression parameters. Country indicators will be included as covariates in multiple country analyses. Further analyses will also evaluate differences in primary characteristics (e.g. cleft type, birth weight) between subjects retained in the study and those who drop out and further adjustment for participation propensity will be applied if real differences emerge.

### Data collection and management

ECLAMC maintains birth records on all participants [18,25]. Updated data on the retrospective group is collected through abstraction of medical records and through phone or home interviews of parents. The pediatricians collect data for the prospective group at birth and at the periodic follow up evaluations in the neonatal and two-year subprojects. Specific data collection forms and outcome measures were developed for the purposes of this study. Samples of data forms are available from the authors on request. Data are entered into personal digital assistants (PDAs) in a system designed and implemented by the Global Network for Women's and Children's Health Research Data Coordinating Center at RTI. Data are transferred on a routine basis from participating hospitals to the study database server in Buenos Aires, and then to RTI via the Internet in a secure, encrypted file with personal identifiers stripped. RTI routinely reports to the National Institute of Child Health and Development (NICHD) appointed Data Monitoring Committee that oversees study progress and outcomes.

### Personnel training

In order to prepare the pediatricians from ECLAMC for participation in the study, training sessions were carried out mostly in conjunction with the annual ECLAMC meetings. In addition to study design and procedures, focused training was provided for the use of PDAs for data collection and for administration of the developmental instruments (BINS and REEL). Both the BINS and the REEL were instruments not commonly used in South America. Following approval from the commercial providers, both were translated and back translated into Spanish and Portuguese. A three-day instructional seminar on the use of the BINS and REEL included power point presentations, training videotapes, practice sessions and written instructions. These instruments were enthusiastically received by participating pediatricians and there have been few concerns expressed over their implementation. We are currently using English language norms for comparison but are working on developing both Spanish and Portuguese versions of the BINS. Training in the use of the PDAs as well as in methods of electronic data transfer provided individualized tutoring in software/hardware use based on the needs and experience of each individual. Retraining is carried out on an annual basis with a primary focus on new and updated procedures.

### Informed consents

Signed informed consent protocols were established after several iterations with local, Iowa, and RTI Institutional Review Boards (IRBs) that included translations and back translations from Spanish and Portuguese to English. Separate signed informed consents are being utilized for the neonatal and two-year subprojects among the prospective group. In case of illiteracy, ECLAMC pediatricians read the informed consent and fully explain its content to the mother or legal guardian of the eligible infant. Confirmation is obtained via thumbprints in this case in the presence of a witness. For the retrospective data collection, verbal consent is obtained from the parent or legal guardian for participation.

## Discussion

This paper describes the methods of a study that is evaluating the effectiveness of a model of care that pediatricians, even in difficult settings, may provide to improve survival and health of children born with birth defects. We believe it is important to document the feasibility of conducting international collaborative clinical trials aimed at prevention and treatment of craniofacial anomalies and other anomalies as strategies to decrease the global burden imposed by birth defects. The intervention in this project is consistent with recommendations of the Institute of Medicine (IOM) to reduce the impact of birth defects in the developing world by providing better treatments for affected children [26]. The IOM report highlights the importance of early thorough assessments to better identify existing anomalies and define the treatment plans for children with birth defects.

Isolated forms of oral clefts pose larger risks for morbidity and challenged development than for mortality among affected infants and children. It is therefore expected that the pediatric intervention may be more effective in improving these outcomes than in reducing the mortality risk in this group.

This study is readily expandable to include infants born with other potentially lethal anomalies that can benefit from early recognition and referral. Neural tube defects and congenital heart disease would be among the common non-cleft birth defects where early intervention may prove life saving until appropriate surgical or medical care can be completed. Furthermore, this intervention may be studied among infants born preterm and/or at low birth weight. Up to 28% of worldwide neonatal deaths are attributed to prematurity, and up to 80% of neonatal deaths occur among children born at low birth weight [3,4], a highly prevalent condition specifically in developing countries where 16% of live births are underweight [27], forming about 95% of worldwide underweight births. Other prevention based approaches such as the folic acid food fortification program in Chile that has decreased neural tube defects by at least 30% in the last three years [28] should be used in conjunction with programs like the one described here that addresses the needs of infants born with birth defects.

The study is being successfully implemented using the preexisting infrastructure of the ECLAMC birth defects surveillance program. The study implementation strategies have a relatively low cost as they rely on an existing infrastructure and a group of dedicated physicians and their colleagues. This approach will easily be transportable to other sites and hypotheses. In future studies we hope to include parents of affected children as members of peer support groups. They can serve as physician extenders to enhance attendance at interventional and follow-up visits and to sustain the intervention in the community if it proves to be effective. Since the studied interventions are of tolerable cost and use existing personnel, the long-term prospects are good for continuing and expanding these research efforts.

## Competing interests

The author(s) declare that they have no competing interests.

## Authors' contributions

All authors have participated in drafting the manuscript and/or thorough review and revision and have approved submitting this version. GW has contributed to study design and procedures and development of data collection instruments. EC has substantially contributed to all aspects of study design, methodology, and implementation. NG has contributed to study design and procedures and development of data collection instruments. MR, VC, GD, JC, and IO have contributed to development of study procedures and data collection instruments and coordination. LJ has contributed to study design and procedures and development of ethics protocols. A McCarthy has contributed to study design and outcome measurement. GB has contributed to study design. SL and A Mariona have contributed to designing and managing the electronic data collection and transmission system. JM has substantially contributed to all aspects of study design, methodology, and implementation. JM and EC conceived the study.

**Table 3:** Study Pediatricians

**Responsible professional**	**City**	**Country**
Rittler, Mónica	Buenos Aires	Argentina
Rottenberg, Daniela	Buenos Aires	Argentina
Cosentino, Viviana	Buenos Aires	Argentina
Jewtuszyk, Mónica	Buenos Aires	Argentina
Lerner, Mario	Gualeguaychú	Argentina
Mussi, Margarita	Rosario	Argentina
Ermini, Mónica	La Plata	Argentina
Cárpena, Luisa	Córdoba	Argentina
Chirino, Andrea	Córdoba	Argentina
Echegaray, Adriana	Córdoba	Argentina
Negri, Carlos	San Martín	Argentina
Menzio, Mónica	San Luis	Argentina
Saleme, César	Tucumán	Argentina
Deguer, Carlos	Bahía Blanca	Argentina
Lombardelli, Rodolfo	Esquel	Argentina
Mereb, Juan Carlos	El Bolsón	Argentina
Rueda, Saúl	La Paz	Bolivia
Nogueira, Áurea	Florianópolis	Brazil
Canonaco, Rosane	Sao Paulo	Brazil
Leite, Julio César	Porto Alegre	Brazil
Cavalcanti, Denise	Campinas	Brazil
Ternes-Pereira, Eliana	Florianópolis	Brazil
Abath, Cristina	Joao Pessoa	Brazil
Acosta, Angelina	Salvador	Brazil
Nazer-Herrera, Julio	Santiago	Chile
Ojeda, María Elena	Rancagua	Chile
Canessa, Aurora	Linares	Chile
Wettig, Elisabeth	Puerto Montt	Chile
Mellado, Cecilia	Santiago	Chile
Farfán, Victor	Talca	Chile
Díaz, Marcela	Santiago	Chile
Zarante, Ignacio	Bogotá	Colombia
García, Natalia	Bogotá	Colombia
Villegas, Carlos A.	Manizales	Colombia
Luna Ballén, Ana M.	La Mesa	Colombia
Cristancho, Camilo	Ubaté	Colombia
Montalvo, Germán	Quito	Ecuador
Toscano, Mario	Manabi	Ecuador
Girón, Cecibel	Manabi	Ecuador
Camacho, Antonio	Ibarra	Ecuador
Sacoto, Adriana	Cañar	Ecuador
Martínez, Ernesto	Azogues	Ecuador
Cedeño, Rosa	Maracaibo	Venezuela
Jatar Senior, Braulio	Coro	Venezuela

## Pre-publication history

The pre-publication history for this paper can be accessed here:


